# Galectin-3: an immune checkpoint target for musculoskeletal tumor patients

**DOI:** 10.1007/s10555-020-09932-4

**Published:** 2020-09-14

**Authors:** Kosei Nakajima, Vitaly Balan, Avraham Raz

**Affiliations:** 1 Division of Translational Research, Exploratory Oncology Research & Clinical Trial Center, National Cancer Center Research Institute, 5-1-1 Tsukiji, Chuo-Ku, Tokyo, 104-0045 Japan; 2 Division of Veterinary Oncology and Surgery, Faculty of Veterinary Medicine, Imabari Campus, Okayama University of Science, 1-3 Ikoinooka, Imabari, Ehime 794-8555 Japan; 3 Refuge Biotechnology, 1505 Adams Dr, Suite D, Menlo Park, CA 94025 USA; 4 Departments of Oncology and Pathology, Barbara Ann Karmanos Cancer Institute, 4100 John R St, Detroit, MI 48201 USA

**Keywords:** Musculoskeletal tumors, Galectin-3, Immune checkpoint inhibitor, Bone immunological microenvironment

## Abstract

In the past decade, the development of immune checkpoint inhibitors in oncological clinical settings was in the forefront. However, the interest in musculoskeletal tumor patients as candidates for checkpoint inhibition remains underserved. Here, we are forwarding evidence proposing that galectin-3 (Gal-3) is an additional immune factor in the checkpoint processes. This review is the result of a large-scale cohort study depicting that overexpression of Gal-3 was widely prevalent in patients with musculoskeletal tumors, whereas T cell infiltrations were generally suppressed in the tumor microenvironment. Targeting Gal-3 would serve as a novel immune checkpoint inhibitor candidate in patients afflicted with aggressive musculoskeletal tumors.

## Galectin-3 discovery

In the early 1980s, limited biochemical differences among tumor cells with low or high metastatic potentials were reported, and the specific surface characteristics associated with metastasis were still undefined. It was shown that tumor cell aggregation and pulmonary metastasis can correlate with distribution of cell surface dense anionic sites [1]. The following year, in 1981, a simple sugar, galactose was reported to inhibit the formation of tumor emboli, leading to the notion that tumor cells express galactose-binding proteins, i.e., lectin. The carbohydrate-binding protein(s) on the surface of malignant cells had been implicated in tumor aggressive behaviors. At that time, it was termed ‘galactoside-specific lectin’ [2]. In 1994, the protein was firstly named ‘galectin-3’ (Gal-3) [3]. Since then, identification studies have clarified that the molecule was also known as IgE-binding protein, MAC2, L-29, CPB-35, *etc.*, since the names had not been organized at that time. Additionally, 14 other galectins were discovered and the family has been classified into three groups according to their structure: (1) prototypical, (2) tandem repeat, and (3) chimeric. In human cells, Gal-1-Gal-15 all express an evolutionarily conserved carbohydrate recognition domain (CRD) that interacts with various glycoproteins containing terminal galactoside residues [4]. One of them, e.g., Gal-3, was found to be a pleiotropic-pluripotent molecule. Presently, consensus is that Gal-3 works as a key driver of tumor progression and is considered a promising therapeutic target.

## Galectin-3 in musculoskeletal tumors

In a recent large-scale cohort study, we show the Gal-3 expression profile in patients with musculoskeletal tumors. As a result, Gal-3 was found to be highly expressed in Ewing’s sarcoma, bone metastasis of breast cancer, a giant cell tumor of bone, as compared with normal bone. In addition, higher expressions were observed in rhabdomyosarcoma, fibrosarcoma, and angiosarcoma. In certain patients with osteosarcoma, undifferentiated pleomorphic sarcoma, chondrosarcoma, chordoma, synovial sarcoma, liposarcoma, leiomyosarcoma, and hemangiopericytoma, Gal-3 expression was at relatively high levels [5]. Thus, the data revealed that Gal-3 expressions were widely prevalent in musculoskeletal tumors.

In light of the molecular function of Gal-3, it tends to approach with other glycosylated proteins rather than taking independent action and thereafter transforms into a malignant feature. For example, Gal-3 interacts with other apoptosis-associated proteins such as Nucling, Synexin, Bax, and FasR (CD95), leading to apoptosis-resistant phenotypes. Furthermore, Gal-3 also plays a significant role as a modulator of major signaling pathways, such as Wnt signaling, Ras/Raf/MAPK signaling, and PI3K/AKT signaling through bindings with β-catenin, K-Ras, and AKT, respectively, which could induce dynamic changes in malignant phenotype. Gal-3 is mainly a cytosolic protein; however, it can translocate into the nucleus by binding with Impotin, Sufu, and Nup98, wherein it controls the cell cycle through the interaction with cyclin A, cyclin D, cyclin E, p21(WAF1), and p27 (KIP1), accelerating cancer cells’ proliferation. Gal-3 transmigrates in the cytoplasm, nucleus, and on the cell surface; thus, Gal-3 shows unique localization [4]. Furthermore, Gal-3 is transported *via* an unknown non-classical pathway into the extracellular milieu *via* vesicular release, exosomal secretion, and traverse lipid bilayer membrane. Due to the function of CRD, Gal-3 binds with numerous proteins, more than 50 molecules as we systematically reviewed [4]. Depending on the localization, Gal-3 accelerates malignancy in musculoskeletal tumors.

In patients with osteosarcoma, a higher expression of Gal-3 was reported to be positively correlated with advanced stage [6], since cytoplasmic Gal-3 enhances the malignant phenotype of osteosarcoma [7, 8]. Osteosarcoma cells are capable of secreting Gal-3 [9], and tumor-secreted Gal-3 influences osteoblasts and osteoclasts; Gal-3 inhibits osteoblast differentiation by Notch signaling activation [10] and mediates osteoclast fusion by interaction with Myosin-2A, a modulator of osteoclast differentiation, accelerating osteolytic bone remodeling [11]. Thus, tumor-secreting Gal-3 drives bone destruction. In addition to tumor cells, Gal-3-positive osteoclast precursors appear to congregate near the matured osteoclasts in the osteosarcoma microenvironment [11]. Thus, Gal-3 enhances the progression of osteosarcoma.

In breast cancer bone metastasis, Gal-3 demonstrates an osteolytic effect. On the other hand, the cleaved form of Gal-3 is more abundant in prostate cancer bone metastases, and the shift to cleaved Gal-3 attenuates the osteoclast differentiation [11]. Of note, during the cancer dissemination process, prostate cancer cells preferentially adhere to human bone marrow endothelium through Gal-3 interaction, enhancing the bone metastasis [12]. Consistently, Gal-3 antibody or lactulose-L-leucine, an inhibitor of Gal-3, suppressed skeletal metastasis in mouse models [13, 14]. These studies indicate that Gal-3 is essential during the bone dissemination, and therefore, targeting Gal-3 may preclude malignant cell lodging in bone metastasis.

In Ewing’s sarcoma, the Gal-3 expression level has been reported approximately 14-fold higher in comparison with that of osteosarcoma [15], suggesting a role of more aggressive and bone destructive clinical behavior. We also observed the potent Gal-3 expressions on the membrane of Ewing’s sarcoma cells [data not shown], implying the functional role of tumor aggregation or adhesion. It should be also noted that previous reports showed that Ewing’s sarcoma secrete exosomes including galectin-3-binding protein (LGALS3BP) [16], and it may be a candidate of favorable prognostic indicator [17], as neutralizing the molecular function of Gal-3. These evidences imply that inhibition of Gal-3 may suppress the aggressive behavior of Ewing’s sarcoma.

Giant cell tumor of bone, an osteoclast-producing tumor, is characterized by osteoclastogenic stromal cells and giant cells, which are excessively multinucleated osteoclast cells. Gal-3-expressing cells were detected in the vicinity of giant cells, and some of the giant cells expressed Gal-3, suggesting a role of osteoclastogenesis [11].

Chordoma often arises from notochord, whereby common locations of the lesion are sacrum and shows bone destructive behavior. Previous study indicated that 75–100% of chordoma were positive for Gal-3 [18, 19]. Considering the frequency of expression, Gal-3 could contribute to tumor malignancy of chordoma.

In malignant soft tissue sarcomas, Gal-3 is widely prevalent, which is supported by a previous reports that have identified Gal-3 expression in fibrosarcoma, chondrosarcoma, undifferentiated pleomorphic sarcoma, liposarcoma, leiomyosarcoma, rhabdomyosarcoma, angiosarcoma, synovial sarcoma to name but a few [20, 21]. Although the Gal-3 function(s) in most soft tissue sarcoma is yet to be established, it should be emphasized that Gal-3 inhibition suppresses angiosarcoma proliferation *in vitro* [22], implying that Gal-3 may contribute to malignant phenotype of soft tissue sarcoma.

## Galectin-3, an immune checkpoint molecule

**Fig. 1:**
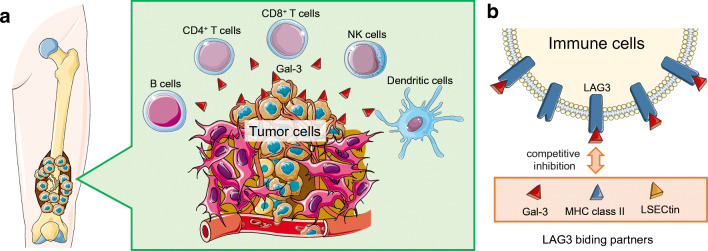
**a** Musculoskeletal tumors express Gal-3 on the cell surface or secrete Gal-3 in extracellular *milieu*, leading to protection from immune cells. **b** Competitive inhibition of LAG3 with binding partners. Immune cells including CD4^+^ T cells, CD8^+^ T cells, natural killer (NK) cells, dendritic cells, and B cells express LAG3. The LAG3 binding with MHC class II and/or LSECtin activates the function of immune cells, whereby Gal-3 interferes with these interactions. The figure was produced using Servier Medical Art with permission

In order to combat the aggressive behavior of musculoskeletal tumors, immunotherapy has been utilized. As for clinical trials for soft tissue sarcoma, osteosarcoma, bone metastasis, and other musculoskeletal tumors, immunotherapies are ongoing in phase I–II, involving immune checkpoint inhibitors, cancer vaccines, adoptive cell therapies, and chimeric antigen receptor T cell (CAR-T) therapy [23–27]. A clinical guideline has recommended pembrolizumab, a programmed cell death protein 1 (PD1) blocking antibody, only in alveolar soft part sarcoma, with evidence of efficacy. However, other checkpoint inhibitors have shown uncertain results in soft tissue sarcomas [28]. Thus, in the past decade, numerous immunotherapeutic modalities have been developed to overcome malignant musculoskeletal tumors; however, several attempts have failed to result in beneficial clinical outcomes with no underlying rationale determined as we mentioned previously [29]. To overcome these challenges, a novel checkpoint target is crucial (Fig. 1a, b).

**Fig. 2:**
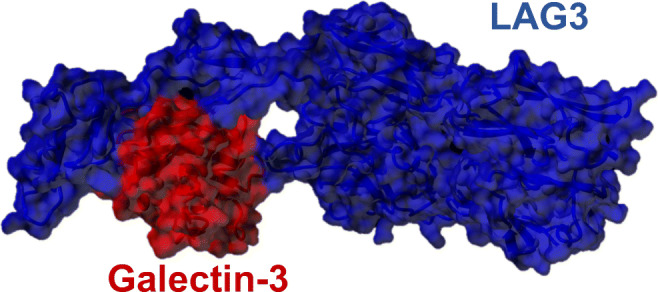
The molecular image represents docking of LAG3 (blue) and CRD domain of Gal-3 (red). Since crystal structure of LAG3 is not known, we performed homology modeling of this protein using YASARA software, a molecular graphic modeling and simulation program

Gal-3-expressing tumor environments are quite distinctive, showing various phenotypic alteration, e.g., escape from immune attacks. Specifically, Gal-3 binds with T cell surface receptors, CD7 and CD29, inducing apoptosis *via* mitochondrial cytochrome c release and caspase-3 activation [30]. Similarly, Gal-3 binds to a complement of T cell surface glycoprotein receptors CD45 and CD71, leading to induce T cell death [31]. Further, Gal-3 promotes TCR downregulation, which inhibits T cell activation and functions [32]. Most importantly, Gal-3 is capable of binding to lymphocyte-activation gene 3 (LAG3), which is necessary for activation CD8^+^ T cells [33–35]. This interaction was confirmed by immunoprecipitation. Further, in order to visualize this finding, we performed homology modeling of LAG3 and docked it with CRD of Gal-3 (Fig. 2). LAG3 belongs to the immunoglobulin superfamily and comprises a 503-amino acid type I transmembrane protein, in which MHC class II and LSECtin bind the extracellular domain of LAG3 [36, 37], whereby Gal-3 interferes the interactions [33]. These evidences suggest that Gal-3 plays a crucial role in the immune checkpoint, and the notion leads to the clinical significance that suppression of Gal-3 enhances the tumor-specific immune response.

## Galectin-3 interference of T cells’ function in musculoskeletal tumor patients

In a big data study of musculoskeletal patients, we have investigated T cell infiltration status, and the result suggested that CD3^+^ T cells and CD8^+^ T cells were predominantly suppressed in bone tumors. CD4^+^ T cells were infiltrated in limited types of tumors including undifferentiated pleomorphic sarcoma, chondrosarcoma, and giant cell tumor of bone, whereas other tumors demonstrated relatively lower infiltration. Similarly, regarding soft tissue sarcoma, this T cell infiltration was largely decreased. These results suggested that T cell function is fundamentally suppressed in the immunological microenvironment of musculoskeletal tumors [38]. The data imply that the immune checkpoint of T cells’ function can be inhibited by tumor-derived Gal-3 in the musculoskeletal tumor microenvironment.

Also, our prospective clinical study showed that the Gal-3 level of patient serum is positively associated with a clinical time course of tumor progression [39]. The finding can be interpretative that the tumor-secreted Gal-3, at least in part, may interfere with the T cell function in the circulation and/or tumor microenvironment. Simultaneously, since cancer patients produce autoantibodies to Gal-3 [39], Gal-3 hinders cancer detection/recognition by the endogenous antibodies and/or immunotherapeutic agents, termed the phenomena as “cancer stealth” effect [29]. These events may result in T cell function interference serving as immune checkpoints (Fig. 1a, b).

## Pharmacological development of targeting galectin-3

**Fig. 3:**
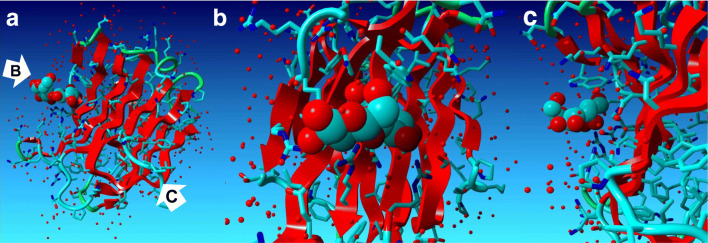
**a** Whole molecular structure of Gal-3 CRD in combination with lactose. The molecular image was depicted with the data of Protein Data Bank #4R9B using YASARA software. **b** An enlarged image of CRD pocket of Gal-3. The amino acid sequence of 158–175 (HFNPRFNENNRRVIVCNT) generates potential hydrogen bonds and van der Waals contacts. **c** Another enlarged view of CRD pocket composed by β sheet structures in line

**Fig. 4:**
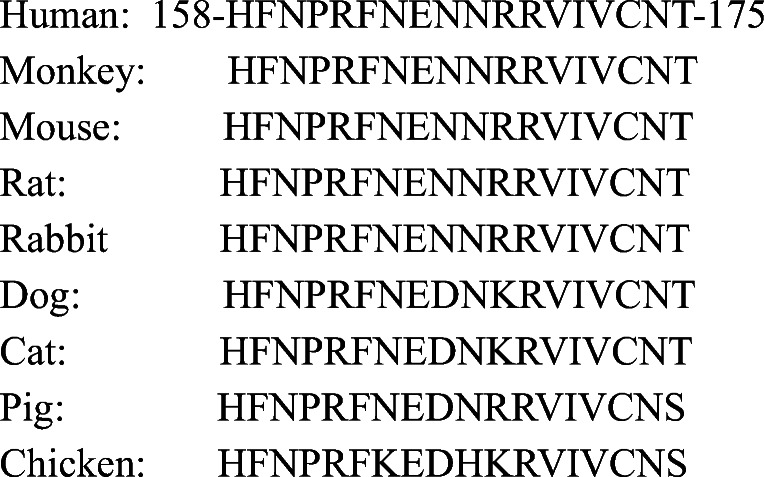
The amino acid sequence of 158–175 in human Gal-3 CRD is evolutionarily conserved among the animal species. The homology search was performed by ClustalW

To date, no clinically available agents specifically inhibiting Gal-3 have been reported, although some *in vivo* studies reported that modified citrus pectin (MCP; A.K.A. GCS-100)) inhibits tumor progression [40]. Another Gal-3 inhibitor, GR-MD-02 (belapectin; galactoarabino-rhamnogalacturonate), is currently being researched in a clinical trial for therapeutic function for nonalcoholic steatohepatitis, severe plaque psoriasis, and metastatic melanoma [41–44]. GCS-100 was tested in relapsed chronic lymphocytic leukemia [45] and refractory solid tumors [46], while similarly, MCP was tested in relapsed prostate cancer [47]. These results may explain the “cancer stealth” effects by tumor-secreting Gal-3 or consumption of the inhibitors due to unspecific binding to other Galectins, in which we proposed that a larger dose of specific Gal-3 inhibitor may resolve the clinical dilemma. Hence, we attempt to target the 158–175 amino acid sequence (HFNPRFNENNRRVIVCNT) in the CRD of Gal-3, which is responsible for its function based on the 3D structure [48] (Fig. 3). Of note, the sequence is evolutionarily conserved among the animal species, suggesting the significance of translational meaning (Fig. 4). Notably, endogenously produced Gal-3 autoantibody in cancer patient does not recognize the CRD [11, 49]. Therefore, we have generated monoclonal neutralizing antibody against the sequence in order to inhibit the Gal-3 function, and it could be utilized as immune checkpoint inhibitor to Gal-3. Thus, suppressing Gal-3 is emerging as one of the immune checkpoint blockage therapies [50].

In conclusion, Gal-3 serves as an immune checkpoint, whereby targeting Gal-3 may suppress the aggressive potential of malignant musculoskeletal tumors.
